# Association of Provider Perspectives on Race and Racial Health Care Disparities with Patient Perceptions of Care and Health Outcomes

**DOI:** 10.1089/heq.2021.0018

**Published:** 2021-07-05

**Authors:** Olivia M. Lin, Hadley W. Reid, Rebecca L. Fabbro, Kimberly S. Johnson, Bryan C. Batch, Maren K. Olsen, Roland A. Matsouaka, Linda L. Sanders, Sangyun Tyler Chung, Laura P. Svetkey

**Affiliations:** ^1^ Duke University School of Medicine, Durham, North Carolina, USA.; ^2^ Division of Geriatrics, Department of Medicine, Duke University School of Medicine Durham, North Carolina, USA.; ^3^ Center for Aging and Human Development, Duke University School of Medicine, Durham, North Carolina, USA.; ^4^ Geriatrics Research Education and Clinical Center, Durham Veterans Affairs Medical Center, Durham, North Carolina, USA.; ^5^ Division of Endocrinology, Department of Medicine, Duke University School of Medicine, Durham, North Carolina, USA.; ^6^ Department of Biostatistics and Bioinformatics, Duke University, Durham, North Carolina, USA.; ^7^ Durham Veterans Affairs Medical Center, Durham, North Carolina, USA.; ^8^ Division of General Internal Medicine, Department of Medicine, Duke University School of Medicine, Durham, North Carolina, USA.; ^9^ Department of Biostatistics and Bioinformatics, Duke University, Durham, North Carolina, USA.; ^10^ Division of Nephrology, Department of Medicine, Duke University School of Medicine, Durham, North Carolina, USA.

**Keywords:** health disparities, diabetes, racial bias, shared decision-making, provider communication, patient–provider interaction

## Abstract

**Purpose:** Research suggests that providers contribute to racial disparities in health outcomes. Identifying modifiable provider perspectives that are associated with decreased racial disparities will help in the design of effective educational interventions for providers.

**Methods:** This cross-sectional study investigated the association between primary care provider (PCP) perspectives on race and racial disparities with patient outcomes.

**Results:** Study participants included 40 PCPs (70% White, 30% racial minority) caring for 55 patients (45% White, 55% Black) with type 2 diabetes mellitus. Associations of provider perspectives on race and racial disparities with patient variables (Interpersonal Processes of Care [IPC] Survey, which measures patient's ratings of their provider's interpersonal skills; medication adherence; glycemic control) were measured using Spearman correlation coefficients. Results suggest that Black patients of providers who reported greater skill in caring for Black patients had more positive perceptions of care in three of four IPC subdomains (Spearman correlation coefficients of −0.43, 0.44, 0.46, all with *p*<0.05); however, Black patients of providers who believe that racial disparities are highly prevalent had more negative perceptions of care in three of four IPC subdomains (Spearman correlation coefficients of 0.38, −0.53, −0.51, all with *p*<0.05). These same provider characteristics had no correlation with outcomes of medication adherence and hemoglobin A1c (HbA1c) or among White patients.

**Conclusion:** Findings suggest that Black patients of providers who felt better equipped to take care of Black patients had a better experience. Therefore, educational interventions for providers may be most effective if they focus on skill development rather than increasing awareness about racial disparities alone.

## Introduction

Compared to White patients, Black patients have higher rates of type 2 diabetes mellitus (T2DM), higher rates of diabetes related complications, and poorer glycemic control.^1,2^ Various social determinants of health contribute to, but do not fully account for, these disparities.^3,4^ Provider factors may also contribute to these disparities. Compared to White patients, Black patients report poorer measures of interpersonal care, including provider communication, information sharing, and shared decision-making.^5^ In addition, Black patients experience shorter visits and their physicians are more verbally dominant (i.e., higher ratio of physician to patient talk time).^5^ Potential mechanisms for these findings include provider expression of implicit racial bias, defined as unconscious attitudes that contribute to subtle, often nonverbal racial discrimination.^6,7^

One solution considered by health care institutions to alleviate provider implicit bias is through implicit bias and cultural competence training. Two systematic reviews found evidence that cultural competence training raises provider awareness of disparities and increases patient satisfaction; however, there is only scarce low-quality evidence that these trainings actually improve clinical outcomes and patient experience of care.^8,9^ Multiple other studies have highlighted the need to both investigate training efficacy and improve educational training interventions for providers.^10–12^

To determine whether improving provider awareness of and perspectives on race and racial disparities may lead to improved patient outcomes, we investigated the association of provider perspectives (i.e., race-related awareness, beliefs, and self-efficacy) with (1) patient perceptions of care, (2) hemoglobin A1c (HbA1c), and (3) medication adherence. We examined this relationship in a cohort of non-Hispanic Black and non-Hispanic White patients with T2DM and their primary care providers (PCPs). Findings from this study will help identify potential areas of focus that may inform the development of more effective provider educational interventions, with the ultimate goal of alleviating provider contribution to racial disparities in health outcomes.

## Methods

### Study design

This is a cross-sectional study of patients with T2DM and their PCPs conducted between November 21, 2019 and February 18, 2020.

### Setting

This study was conducted in the setting of 23 Duke University Health System-affiliated primary care clinics in North Carolina.

### Participants

This study is part of a larger project, with enrollment of both patients and providers.^13^ In this report, we include the subset of patients whose provider also enrolled in the study and likewise only those providers whose patient(s) also enrolled.

Patient eligibility criteria included (1) Black or White race; (2) non-Hispanic ethnicity; (3) age ≥18 years old; (4) diagnosis of T2DM; (5) >1 HbA1c measurements in the past year; (6) prescribed ≥1 daily antihyperglycemic medication; and (7) >1 primary care clinic visit in the past year with the same PCP, associated with an ICD 10 code of T2DM. All eligibility criteria were assessed using Duke Enterprise Data Unified Content Explorer (DEDUCE)^*^ search of the electronic medical record (EMR) within the Duke University Health System. Given an enrollment goal of 200 patients and anticipated response rate of ∼10%, a random sample of 1983 patients was generated from an eligible cohort of 14,847 patients, stratified by provider and race.

Provider eligibility criteria included (1) PCP (physician, nurse practitioner, or physician assistant) at a Duke-affiliated clinic and (2) at least one patient eligible for the study. We identified a total of 394 providers represented among the DEDUCE-generated cohort of 14,847 eligible patients. To minimize nonrandom effects of patient grouping by provider, enrollment was limited to no more than five patients per provider. Thus, given an enrollment goal of 200 patients, an enrollment goal of 40 providers was set. Given an anticipated response rate of ∼10%, all 394 eligible providers were recruited for the study.

### Recruitment

**FIG. 1.**
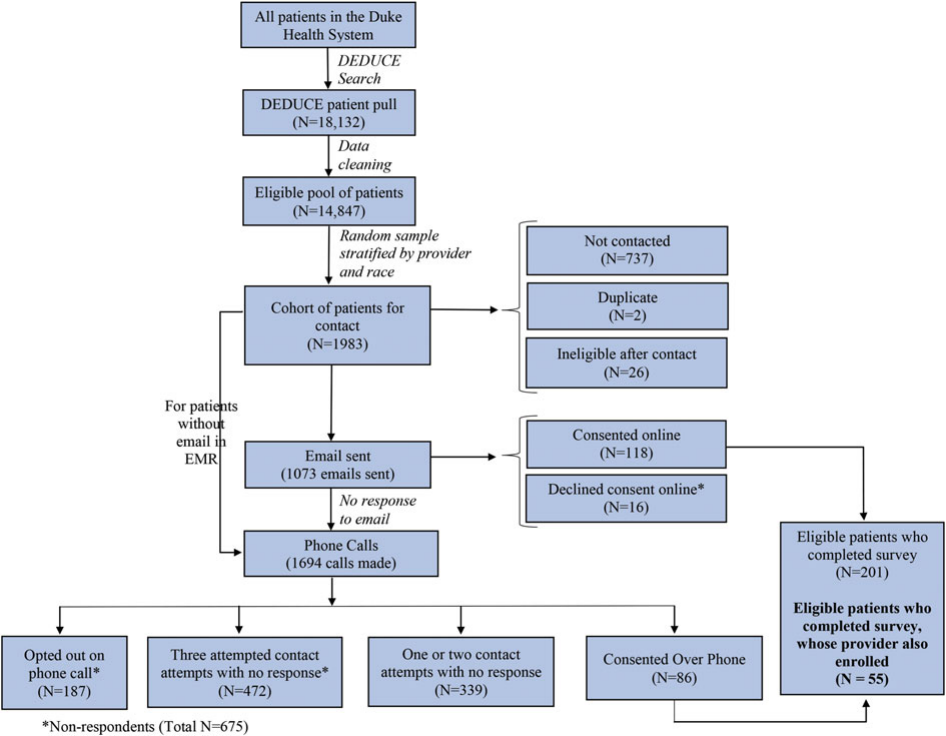
Patient recruitment. This figure describes how the patient study population was recruited and enrolled.

**FIG. 2.**
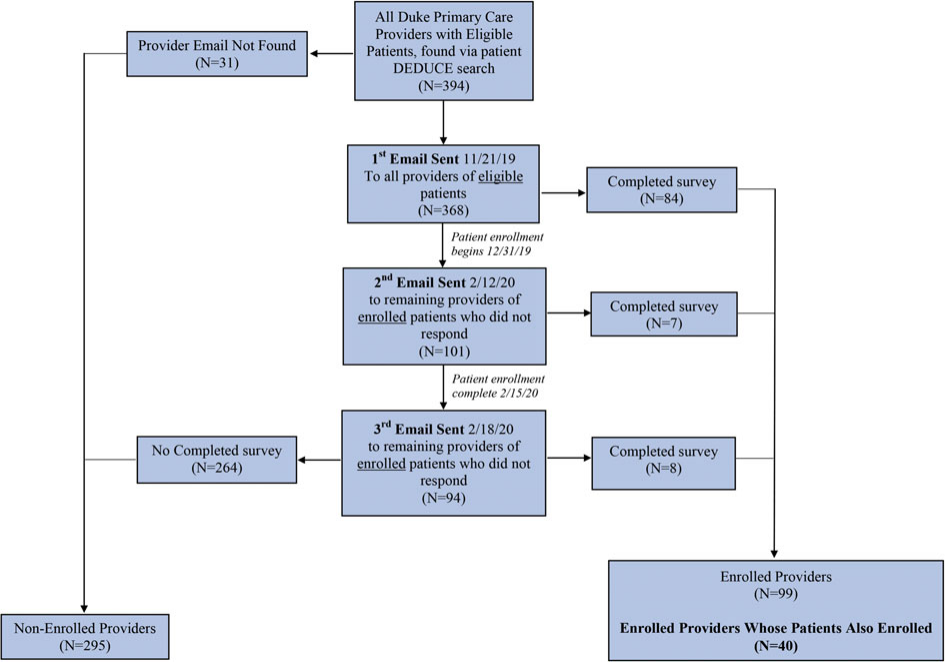
Provider recruitment. This figure describes how the provider study population was recruited and enrolled.

Patients and providers were enrolled through a parallel recruitment process and completed several surveys. All patients provided informed consent before participating. Providers were informed that by completing the online survey, they were consenting to participate. The consent procedures and study protocol were approved by the Duke Institutional Review Board. Patients and providers were able to opt out at any time and were informed that their responses would be confidential. Patients were recruited either through email or phone, and providers were recruited through email. In total, 221 patients and 99 providers enrolled in the study. In this analysis we included the 55 patients whose PCP also enrolled and the 40 PCPs who had 1–3 patient(s) also enroll (Figs. 1 and 2).

### Data sources

#### Provider survey data

Provider enrollees provided demographic information (self-reported gender, race, ethnicity, and clinical information) and completed the Provider Perspectives on Race and Racial Disparities (PPRR) survey, designed to assess provider awareness of and perspectives on race, racism, and racial health care disparities. In the absence of an appropriate existing validated survey, the 9-item PPRR was created based on a literature review by incorporating eight individual items, from three previous studies, that are specifically related to provider awareness of race, racism, and racial health care disparities.^14–16^ Because patients answered questions about perceptions of care through the Interpersonal Processes of Care (IPC), one additional item was included in the PPRR to assess provider awareness of their patients' perceptions of care (Item 9, Table 1). Three subdomains were defined *a priori* based on their related content. For each subdomain, internal consistency was assessed using Cronbach's alpha (CA). Groupings with CA between 0.7 and 0.9 were accepted, as CA <0.7 suggests poor internal consistency and CA >0.9 suggests duplicative items.^17^ CA was in the acceptable range for all but one subdomain, for which CA was subsequently brought into the acceptable range after eliminating one item (Supplementary Table S1); thus the final PPRR survey contains eight items (Table 1). We pilot tested the survey with providers participating in a health disparities research course and made iterative edits to improve clarity based on feedback.

**Table 1.** Provider Perspectives on Race and Racial Disparities Survey Items

Subdomain	Item
(1) Provider Belief^a^	(1) In general (in the United States), how often do you think people with similar medical conditions receive different care based on their race?
	(2) In the clinic where your primary practice is located, how often do you think patients with similar medical conditions receive different care based on their race?
	(3) In your own practice, how often do you think patients with similar medical conditions receive different care based on their race?
(2) Provider Awareness^b^	(4) I am knowledgeable about the historical and contemporary impact of racism, bias, prejudice, and discrimination in health care experienced by various population groups in the United States.^c^
	(5) There is evidence supporting the existence of racial/ethnic disparities in care that are not explained by other factors (e.g., socioeconomic status, education level).
	(6) Being White affords people many privileges in the United States that minorities do not have.
(3) Provider Self-Efficacy^b^	(7) I am as effective at caring for Black patients as I am at caring for White patients.
	(8) I am confident in my ability to provide quality care for Black patients.
	(9) Compared to White patients, Black patients perceive the quality of my care as worse.^d^

^a^ Likert answer choices for items in Subdomain 1: Never, Rarely, Sometimes, Often, Always.

^b^ Likert answer choices for items in Subdomains 2 and 3: Strongly disagree, Disagree, Neither Agree nor Disagree, Agree, Strongly Agree.

^c^ Included *a priori* in Subdomain 2 but eliminated postdata collection after Cronbach's alpha calculation.

^d^ Item reverse scored.

The three subdomains in the PPRR include: (1) Provider Belief (provider belief regarding the prevalence of racial disparities in health outcomes), (2) Provider Awareness (provider awareness of the contemporary impact of racism), and (3) Provider Self-Efficacy (provider confidence and perceived self-efficacy in caring for Black patients). All items are scored on a Likert scale from 1 to 5, with 1 indicating complete disagreement and 5 indicating complete agreement with the statement. Subdomain scores are reported as mean score per question with a score range of 1–5. Higher scores indicate higher belief, awareness, or self-efficacy. Each subdomain is considered an independent variable.

#### Patient survey data

Patient enrollees provided demographic information (self-reported gender, race, ethnicity, and financial security) and completed the IPC Survey and Extent of Adherence Survey.^18,19^ The IPC has been validated in diverse cohorts and used to describe disparities in patient perceptions of medical care.^13^ Because research has shown that providers deliver less effective communication and engage in less shared decision-making with Black versus White patients, IPC subdomains specific to communication and decision-making were included in this analysis.^6^ These subdomains assess patient rating of how often their provider: (1) communicated in a hurried manner; (2) elicited concerns and responded; (3) explained results and medications; and (4) engaged in patient-centered decision-making. For subdomain 1, lower scores indicate higher patient rating of their provider (i.e., “did not hurry often”). For subdomains 2–4, higher scores indicate higher patient rating of their provider (i.e., “explained results often”). Each subdomain is reported as a mean score per question with a score range of 1–5, and each subdomain is considered an independent outcome.

The Extent of Adherence Survey is a three-question survey assessing self-reported medication nonadherence over the past week.^14^ Lower scores indicate higher adherence. Total score is reported as mean score per question with a score range of 1–5.

#### Patient clinical data

We abstracted additional clinical data from the EMR, including age, health insurance, glycemic control (measured through the most recent HbA1c), number and route of antihyperglycemic medications (measured through review of medication list), and comorbidities (measured through review of ICD-10 diagnoses).

### Statistical methods

Demographic characteristics were tabulated for both patient and provider cohorts. Patient outcomes (IPC subdomain, HbA1c, and medication adherence) were summarized both overall and by race. Given the small sample size, this study included both nonparametric and parametric analyses to confirm that the results were robust. For nonparametric analyses, differences in outcomes among Black and White patients were examined through Wilcoxon rank-sum tests. Associations between PPRR subdomain and each outcome were assessed using Spearman's correlation coefficients for the overall patient cohort and stratified by race. For parametric analyses, and to assess whether race moderated the relationship between Provider Awareness, Belief, and Self-efficacy with each outcome, linear regression models with a race interaction term were also conducted. Due to small sample size, patient race was the only covariate included in linear regression models. Wilcoxon rank-sum tests, correlations, and race interaction terms were considered statistically significant if *p*<0.05.

Potential nonrespondent bias was assessed by comparing basic demographic information in nonrespondents to respondents. Nonrespondents were defined as eligible patients who (1) opted out; (2) were not reached with three attempts; (3) declined consent; or (4) began but did not complete consent (Fig. 1). Basic demographic information and patient outcomes for patients whose provider enrolled and thus were included in analysis (*N*=55) versus patients whose provider did not enroll and thus were excluded from analysis (*N*=146) were also compared. All statistical analyses were conducted utilizing SAS, version 9.4.

## Results

Patient nonrespondents and patients excluded from analysis (i.e., whose provider did not also enroll in the study) were similar in age and gender compared to patients included in analysis (Supplementary Table S2). Nonresponse rate among Black and White patients was 72.5% and 62.8%, respectively.

Our analysis included 40 PCPs (26 physicians, 7 nurse practitioners, and 7 physician assistants) who cared for 55 patients with T2DM.

Among patients, 43.6% identified as male, with an average age of 65 years. Most (72.7%) had at least some post high school education, and most (90.9%) had been seeing their PCP for at least 1 year. Thirty (55%) patients identified as Black, and 25 (45%) identified as White. Racial subgroups were similar in demographic characteristics (Table 2). Mean HbA1c was 7.7% among White patients and 8.2% among Black patients, although this difference was not statistically significant (*p*=0.49). In addition, medication adherence did not differ by race, with a mean score of 2.3 for White patients versus 2.5 for Black patients (*p*=0.40; Table 3). Black and White patients were similar in their report of how often their provider communicated in a hurried manner, elicited and responded to concerns, explained results and medications, and engaged in patient-centered decision-making (Table 3).

**Table 2.** Patient Characteristics

	Overall	Black	White
*n* (%)	**55 (100)**	**30 (55)**	**25 (45)**
Age, mean (SD)	65.0 (10.0)	65.1 (8.8)	64.8 (11.3)
Male, *n* (%)	24 (43.6)	10 (33.3)	14 (56.0)
Insurance, *n* (%)			
Medicaid	0 (0)	0 (0)	0 (0)
Medicare	18 (32.7)	8 (26.7)	10 (40.0)
Private	20 (36.4)	12 (40.0)	8 (32.0)
Mixed/both private and public	17 (30.9)	10 (33.3)	7 (28.0)
Financial security,^a^*n* (%)			
High	19 (34.5)	9 (30.0)	10 (40.0)
Medium	17 (30.9)	11 (36.7)	6 (24.0)
Low	18 (32.7)	10 (33.3)	8 (32.0)
Education, *n* (%)			
High school or less	15 (27.3)	10 (33.3)	5 (20.0)
Some postsecondary	22 (40.0)	12 (40.0)	10 (40.0)
Bachelor's degree or greater	18 (32.7)	8 (26.7)	10 (40.0)
Length of provider relationship, *n* (%)			
<1 Year	5 (9.1)	3 (10.0)	2 (8.0)
1–3 Years	20 (36.4)	9 (30.0)	11 (44.0)
>3 Years	30 (54.5)	18 (60.0)	12 (48.0)
Presence of comorbidities, *n* (%)			
Neuropathy	12 (21.8)	5 (16.7)	7 (28.0)
Chronic kidney disease	3 (5.5)	0 (0)	3 (12.0)
Retinopathy	2 (3.6)	0 (0)	2 (8.0)
Previous heart attack	5 (9.1)	3 (10.0)	2 (8.0)
Previous stroke or TIA	12 (21.8)	7 (23.3)	5 (20.0)
Number of antihyperglycemic medications, *n* (%)			
1	23 (41.8)	12 (40.0)	11 (44.0)
2	19 (34.5)	14 (46.7)	5 (20.0)
3+	13 (23.6)	4 (13.3)	9 (36.0)
Route of medication administration, *n* (%)			
Oral	32 (58.2)	15 (50.0)	17 (68.0)
Subcutaneous	4 (7.3)	3 (10.0)	1 (4.0)
Both	19 (34.5)	12 (40.0)	7 (28.0)
No. of appointments with provider in the past year, median (Q1, Q3)	4 (3, 5)	4 (3, 5)	4 (3, 5)

Bold values indicate sample sizes.

^a^ *N*=24 White, 30 Black due to 1 nonresponse among White patients. Self-reported financial security was surveyed and categorized as follows:

(1) After paying the bills you still have enough money for special things that you want. (Categorized as “High” Financial security).

(2) You have enough money to pay the bills, but little spare money to buy extra or special things. (Categorized as “Medium” financial security).

(3) You have money to pay the bills, but only because you have cut back on things. (Categorized as “Low” financial security).

(4) You are having difficulty paying the bills, no matter what you do. (Categorized as “Low” financial security).

SD, standard deviation; TIA, transient ischemic attack.

**Table 3.** Patient Outcome Measures

Outcome^a^	Overall (N=55), mean (SD)	Black (N=30), mean (SD)	White (N=25), mean (SD)	p-Value, Black versus White mean
IPC 1: Hurried communication	1.4 (0.5)	1.3 (0.5)	1.4 (0.5)	0.27
IPC 2: Elicited concerns, responded	4.8 (0.5)	4.9 (0.4)	4.6 (0.5)	0.06
IPC 3: Explained results, medications	4.5 (0.8)	4.5 (0.8)	4.4 (0.8)	0.54
IPC 4: Patient-centered decision-making^b^	4.2 (1.0)	4.2 (0.9)	4.1 (1.1)	0.76
HbA1c	8.0 (1.9)	8.2 (2.2)	7.7 (1.5)	0.49
Medication adherence^c^	2.4 (1.1)	2.5 (1.2)	2.3 (1.1)	0.40

^a^ Each IPC subdomain has a score range of 1–5. For IPC 1, higher score indicates more negative patient perceptions of care (i.e., hurried communication). For IPC 2–4, higher score indicates more positive patient perceptions of care (i.e., decided together). Medication adherence is reported with a score range of 1–5, where lower scores indicate better adherence.

^b^ *N*=54 due to 1 nonresponse among White patients.

^c^ *N*=54 due to 1 nonresponse among Black patients.

HbA1c, hemoglobin A1c; IPC, Interpersonal Processes of Care.

Among PCPs, 67.5% identified as female, 70% identified as White, and 5% identified as Black. Sixty-four percent of providers graduated medical school in 2000 or later, 75% reported at least 25 h of direct patient care per week, and 92.5% reported at least 1 h of training related to implicit bias or a similar topic in the past 5 years (Supplementary Table S3). Twenty-six providers had a single patient enrolled in the study, 13 providers had 2 patients enrolled, and 1 provider had 3 patients enrolled. Out of a possible score of 5 for each scale, mean score among all providers was 2.7 (standard deviation [SD] 0.7) for Provider Belief, 4.0 (SD 0.8) for Provider Awareness, and 4.2 (SD 0.6) for Provider Self-Efficacy; scores appeared to be similar among demographic subgroups (Supplementary Table S3).

### Provider belief versus IPC subdomains

With respect to Provider Belief (provider belief regarding the prevalence of racial disparities in health outcomes), there was surprisingly a negative correlation of −0.28 (*p*=0.036) with the IPC subdomain “Explained results, medications” among patients overall (Table 4). This means that the more prevalent that providers believed racial disparities were, the less frequently patients overall felt their provider explained results and medications.

**Table 4.** Overall Spearman Correlation Coefficients for Provider Perspectives on Race and Racial Disparities Versus Outcomes

	Provider belief	Provider awareness	Provider self-efficacy
IPC 1: Hurried communication	0.12	0.03	−0.22
IPC 2: Elicited concerns, responded	−0.02	−0.06	0.25
IPC 3: Explained results, medications	−0.28^a^	−0.11	0.28^a^
IPC 4: Patient-centered decision-making	−0.25	−0.06	0.23
HbA1c	0.22	0.14	0.02
Medication adherence	0.08	0.04	−0.10

See Supplementary Table S4 for full model results.

^a^ *p*<0.05.

**FIG. 3.**
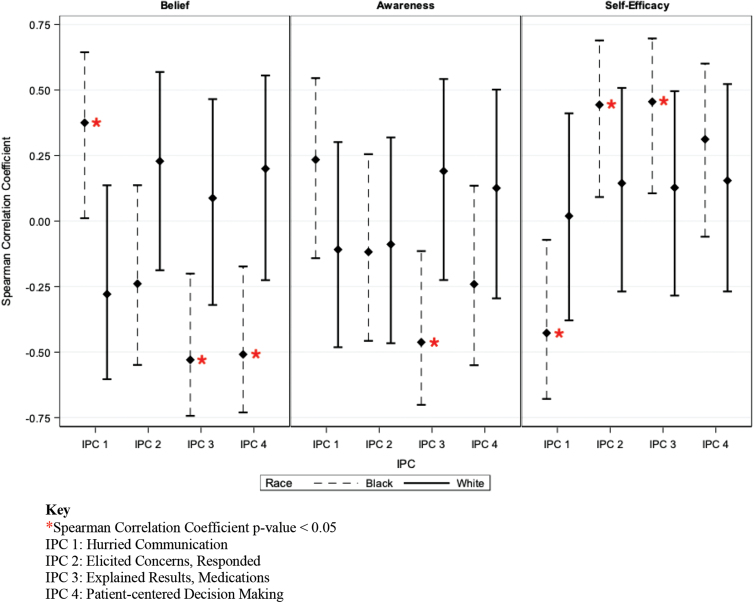
Spearman correlation coefficients of PPRR versus IPC by patient race. This figure compares Black and White confidence intervals for Spearman correlation coefficients of the three PPRR subdomains (Provider Belief, Awareness, and Self-Efficacy) versus four IPC Subdomains (IPC 1–4) by patient race. For IPC 1, a positive correlation coefficient indicates that higher provider Awareness, Belief, or Self-Efficacy is correlated with more negative patient perceptions of care. For IPC 2–4, a positive correlation coefficient indicates that higher provider Awareness, Belief, or Self-Efficacy is correlated with more positive patient perceptions of care. A table of correlation coefficients and *p*-values can be found in Supplementary Table S4. IPC, Interpersonal Processes of Care; PPRR, Provider Perspectives on Race and Racial Disparities.

Among Black patients, there was a positive correlation of 0.38 (*p*=0.040) between Provider Belief and the IPC subdomain “Hurried communication,” a negative correlation of −0.53 (*p*=0.002) between Provider Belief and the IPC subdomain “Explained results, medications,” and a negative correlation of −0.51 (*p*=0.004) between Provider Belief and the IPC subdomain “Patient-centered decision making” (Fig. 3). This means that the more prevalent that providers believed racial disparities were, the more frequently Black patients felt their providers were hurried and the less frequently Black patients felt their providers explained results, explained medications, and engaged in patient-centered decision-making.

### Provider awareness versus IPC subdomains

With respect to Provider Awareness (provider awareness of the contemporary impact of racism), there were no significant correlations with any outcomes in the overall patient population.

Among Black patients, there was a negative correlation of −0.46 (*p*=0.009) with the IPC subdomain “Explained results, medications” (Fig. 3). This means that the more aware providers were of the contemporary impacts of racism, the less frequently Black patients felt their provider explained results and medications.

### Provider self-efficacy versus IPC subdomains

With respect to Provider Self-Efficacy (provider confidence and perceived self-efficacy in caring for Black patients), there was a positive correlation of 0.28 (*p*=0.038) with the IPC subdomain “Explained results, medications” overall. This means that the more confident providers felt about their ability to care for Black patients, the more frequently patients overall felt their provider explained results and medications (Table 4).

Among Black patients, there was a negative correlation of −0.43 (*p*=0.018) between Provider Self-Efficacy and the IPC subdomain “Hurried Communication” and a positive correlation of 0.44 (*p*=0.013) and 0.46 (*p*=0.011) between Provider Self-Efficacy and IPC subdomains “Elicited concerns, responded” and “Explained results, medications,” respectively (Fig. 3). This means that the more confident providers felt about their ability to care for Black patients, the less frequently Black patients felt their providers were hurried and the more frequently Black patients felt their provider explained results, explained medications, and responded to their concerns.

### Results among White patients

No significant correlations were found among White patients in any provider subdomain.

### Additional outcomes of interest

No significant correlations were found for HbA1c and medication adherence outcomes, either overall or by racial subgroup, in any provider subdomain (Table 4 and Supplementary Table S4). No significant race interaction terms were found in the linear regression analyses (Supplementary Table S5).

## Discussion

The aim of this study was to determine whether potentially modifiable provider beliefs, awareness, and self-efficacy with regard to equitable care were associated with (1) patient perception of care and (2) health outcomes. Our main results show that Black patients of providers who believed racial disparities are highly prevalent had more negative perceptions of care; however, Black patients of providers who reported high self-efficacy in caring for Black patients had more positive perceptions of care. There were no significant correlations among White patients, which was expected given the nature of the study questions. Overall, these findings may have implications for developing interventions to mitigate the role of providers in perpetuating disparities.

We were surprised to find that the more prevalent providers believed racial disparities were, the less likely their Black patients were to rate them highly skilled in communication and shared decision-making. Although these findings are exploratory and should be interpreted with caution, several potential explanations exist. A previous study found that providers' self-reported cultural competence behaviors, including explaining medications and fostering clear communication, were not associated with patient report of these behaviors.^20^ This suggests that providers' self-report of cultural competence or related topics may not be correlated with their actual behaviors. Another study found that prejudice-reduction interventions that emphasize external or societal pressure to reduce prejudice may actually increase both implicit and explicit prejudice.^21^ As 92.5% of providers in our sample reported at least 1 h of training related to implicit bias or related topics, it is possible that despite their goal, some provider trainings may include content which could result in provider behaviors that are perceived by patients as being of lower quality. We had no information on the specific content of trainings. Finally, providers have reported in previous studies that increasing awareness alone did not empower them to mitigate the complex causes of racial disparities.^22^ Overall, it may be that to affect patient perceptions of equitable care, increasing awareness among providers needs to be coupled with applicable skill development in domains we studied, including communication and shared decision-making.

Consistent with the idea that skill development may be critical, when providers rated themselves skilled at caring for Black patients, Black patient rating of their provider's interpersonal skills was higher. Assuming that self-efficacy reflects actual level of skill in caring for Black patients, these findings also suggest that provider training may be most effective at alleviating disparities in patient-centered care if focused on skill development.

Our study has several limitations. Given the severity of disparities among Black patients, we deliberately included only non-Hispanic Black and non-Hispanic White patients; consequently, these findings may not be generalizable to other racial or ethnic subgroups. Due to small sample size, we were not able to adjust for the many potentially relevant covariates (e.g., duration of T2DM) in the statistical analyses. Determining the impact of covariates requires further research. Finally, the PPRR is a newly created survey that has not been previously validated. However, in the absence of a similar preexisting tool, this survey was constructed from questions used in previous studies and was pilot tested among providers before use.

## Conclusion

In summary, we conducted a cross-sectional study of patients with T2DM and their PCPs and found that Black patients of providers who believed racial disparities were highly prevalent had more negative perceptions of care in domains of communication and shared decision-making; however, Black patients of providers who reported greater self-efficacy in caring for Black patients had more positive perceptions of care in those same domains. Results suggest that provider trainings may be most effective if they focus on empowering providers with skills to act on information they receive about racial disparities, rather than increasing awareness about racial disparities alone. Future studies of larger, more heterogenous patient populations are needed to determine if educational interventions to improve provider skills in caring for Black patients, targeting communication and shared decision-making, lead to reduced racial disparities in clinical outcomes.

## Supplementary Material

Supplemental data

Supplemental data

Supplemental data

Supplemental data

Supplemental data

## Abbreviations Used

CA: Cronbach's alpha
DEDUCE: Duke Enterprise Data Unified Content Explorer
EMR: electronic medical record
HbA1c: hemoglobin A1c
IPC: Interpersonal Processes of Care
PCP: primary care provider
PPRR: Provider Perspectives on Race and Racial Disparities
SD: standard deviation
T2DM: type 2 diabetes mellitus

## Notes

^*^ A tool available to Duke faculty that enables the quantification of potential study subjects at Duke based on varying inclusion and exclusion criteria available in clinical records.
