# Supplementary material for: Association of Provider Perspectives on Race and Racial Health Care Disparities with Patient Perceptions of Care and Health Outcomes
Source: Health Equity. 2021 Jul 5;5(1):466–75. doi: 10.1089/heq.2021.0018 (PMC8309434; doi:10.1089/heq.2021.0018)
Supplement: Supplemental data [file Supp_Table1.docx]

| **Supplemental Table 1: Cronbach’s Alpha Calculations for PPRR Survey** | |
| --- | --- |
| **PPRR Items** | **Cronbach’s Alpha** |
| 1, 2, 3 | 0.84 |
| 4, 5, 6 | 0.50 |
| 7, 8, 9 | 0.70 |
| 4, 5 | 0.34 |
| 5, 6 | 0.73 |
| 4, 6 | -0.08 |
