# Supplementary material for: Association of Provider Perspectives on Race and Racial Health Care Disparities with Patient Perceptions of Care and Health Outcomes
Source: Health Equity. 2021 Jul 5;5(1):466–75. doi: 10.1089/heq.2021.0018 (PMC8309434; doi:10.1089/heq.2021.0018)
Supplement: Supplemental data [file Supp_Table2.docx]

| **Supplemental Table 2: Analysis of Non-Respondent and Excluded Participant Bias** | | | | | | |
| --- | --- | --- | --- | --- | --- | --- |
|  | **Patient Non-respondents** | | | **Patients Excluded from Analysis^a^** | | |
|  | **Overall (675)** | **Black (301)** | **White (374)** | **Overall (146)** | **Black (71)** | **White (75)** |
| **Age**, Mean (SD) | 65.2 (13.3) | 64.5 (12.9) | 66.1 (13.7) | 64.0 (11.7) | 61.9 (11.7) | 66 (11.3) |
| **Gender,** Male (%) | 304 (45%) | 142 (38.0%) | 162 (53.8%) | 68 (46.6%) | 22 (31.0%) | 46 (61.3%) |
| **HbA1c,** Mean (SD) | 7.4 (1.4) | 7.5 (1.5) | 7.3 (1.3) | 7.2 (1.2) | 7.3 (1.3) | 7.2 (1.1) |
| 1. Enrolled patients whose provider did not also enroll in the study were excluded from analysis | | | | | | |
