# Supplementary material for: Association of Provider Perspectives on Race and Racial Health Care Disparities with Patient Perceptions of Care and Health Outcomes
Source: Health Equity. 2021 Jul 5;5(1):466–75. doi: 10.1089/heq.2021.0018 (PMC8309434; doi:10.1089/heq.2021.0018)
Supplement: Supplemental data [file Supp_Table3.docx]

| **Supplemental Table 3: Mean PPRR scores by Provider Subgroup** | | | | |
| --- | --- | --- | --- | --- |
|  | **N (%)** | **Provider**  **Belief**  Mean score (SD) | **Provider Awareness**  Mean score (SD) | **Provider Self-Efficacy**  Mean score (SD) |
| **Overall** | 40 (100%) | 2.7 (0.7) | 4.0 (0.8) | 4.2 (0.6) |
| **Type of Provider** |  |  |  |  |
| Physician (MD) | 24 (60.0%) | 2.9 (0.7) | 4.2 (0.8) | 4.1 (0.6) |
| Physician (DO) | 2 (5.0%) | 2.8 (0.7) | 3.5 (0.7) | 4.0 (0.5) |
| Nurse Practitioner | 7 (17.5%) | 2.3 (0.7) | 3.9 (0.7) | 4.3 (0.5) |
| Physician Assistant | 7 (17.5%) | 2.2 (0.6) | 3.6 (0.7) | 4.7 (0.4) |
| **Gender** |  |  |  |  |
| Male | 13 (32.5%) | 2.9 (0.7) | 4.3 (0.7) | 4.1 (0.8) |
| Female | 27 (67.5%) | 2.6 (0.7) | 3.9 (0.8) | 4.3 (0.5) |
| **Race** |  |  |  |  |
| White | 28 (70.0%) | 2.7 (0.8) | 4.0 (0.9) | 4.2 (0.7) |
| Black or African American | 2 (5.0%) | 3.2 (0.7) | 4.5 (0.0) | 4.3 (0.9) |
| Asian | 5 (12.5%) | 2.7 (0.7) | 3.9 (0.2) | 4.5 (0.5) |
| American Indian or Alaska Native *or* Native Hawaiian or Pacific Islander | 2 (5.0%) | 2.3 (0.9) | 3.5 (0.7) | 4.0 (0.0) |
| Another race | 3 (7.5%) | 2.6 (0.4) | 4.2 (1.0) | 4.1 (0.5) |
| **Ethnicity^a^** |  |  |  |  |
| Non-Hispanic/Latinx | 38 (95.0%) | 2.7 (0.7) | 4.0 (0.8) | 4.2 (0.6) |
| Prefer not to answer | 1 (2.5%) | 3.0 | 5.0 | 4.0 |
| Missing | 1 (2.5%) | 2.7 | 3.5 | 4.3 |
| **Year of medical school graduation** |  |  |  |  |
| Earlier than 2000 | 14 (35.0%) | 2.7 (0.8) | 3.9 (1.0) | 4.3 (0.5) |
| 2000-2009 | 10 (25.0%) | 2.7 (0.6) | 4.2 (0.7) | 4.1 (0.5) |
| 2010-2019 | 15 (37.5%) | 2.7 (0.8) | 4.0 (0.8) | 4.2 (0.8) |
| **Direct Patient Care Hours per Week^b^** |  |  |  |  |
| 10-24 | 9 (22.5%) | 2.7 (0.9) | 4.2 (0.6) | 4.4 (0.6) |
| 25-34 | 9 (22.5%) | 2.8 (0.6) | 4.1 (0.5) | 3.9 (0.3) |
| 35-39 | 16 (40.0%) | 2.6 (0.6) | 3.9 (1.0) | 4.5 (0.5) |
| 40+ | 5 (12.5%) | 2.8 (1.0) | 4.1 (1.0) | 3.7 (1.0) |
| **Provider Hours of Anti-bias Training^c^** |  |  |  |  |
| 0 | 2 (5.0%) | 2.7 (0.9) | 4.0 (0.0) | 4.5 (0.7) |
| 1-2 | 21 (52.5%) | 2.5 (0.7) | 3.7 (0.8) | 4.3 (0.6) |
| 3-4 | 10 (25.0%) | 3.0 (0.7) | 4.5 (0.6) | 4.0 (0.6) |
| 5+ | 6 (15.0%) | 2.9 (0.8) | 4.3 (0.9) | 4.1 (0.8) |
