# Supplementary material for: Association of Provider Perspectives on Race and Racial Health Care Disparities with Patient Perceptions of Care and Health Outcomes
Source: Health Equity. 2021 Jul 5;5(1):466–75. doi: 10.1089/heq.2021.0018 (PMC8309434; doi:10.1089/heq.2021.0018)
Supplement: Supplemental data [file Supp_Table4.docx]

| **Supplemental Table 4: Correlation between PPRR and Outcomes: Full Model Results^a^** | | | | | | | | |
| --- | --- | --- | --- | --- | --- | --- | --- | --- |
|  | **Provider Belief** | | | | | | | |
|  | **Overall (N=55)** | | **Black (N=30)** | | | **White (N=25)** | | |
|  | **Spearman** | **p-value** | **Spearman** | **95% CI** | **p-value** | **Spearman** | **95% CI** | **p-value** |
| **IPC 1: Hurried communication** | 0.12 | 0.37 | 0.38 | (0.01, 0.64) | 0.040 | -0.28 | (-0.60, 0.14) | 0.18 |
| **IPC 2: Elicited concerns, responded** | -0.02 | 0.91 | -0.24 | (-0.55, 0.14) | 0.21 | 0.23 | (-0.19, 0.57) | 0.28 |
| **IPC 3: Explained results, medications** | -0.28 | 0.036 | -0.53 | (-0.74, -0.20) | 0.002 | 0.09 | (-0.32, 0.47) | 0.68 |
| **IPC 4: Patient-centered decision making^b^** | -0.25 | 0.07 | -0.51 | (-0.73, -0.17) | 0.004 | 0.20 | (-0.23, 0.56) | 0.35 |
| **HbA1c** | 0.22 | 0.10 | 0.29 | (-0.08, 0.59) | 0.12 | 0.10 | (-0.31, 0.47) | 0.65 |
| **Medication Adherence** | 0.08 | 0.54 | 0.17 | (-0.21, 0.50) | 0.38 | -0.18 | (-0.53, 0.24) | 0.40 |
|  | **Provider Awareness** | | | | | | | |
| **IPC 1: Hurried communication** | 0.03 | 0.81 | 0.23 | (-0.14, 0.55) | 0.22 | -0.11 | (-0.48, 0.30) | 0.61 |
| **IPC 2: Elicited concerns, responded** | -0.06 | 0.67 | -0.12 | (-0.46, 0.26) | 0.54 | -0.09 | (-0.47, 0.32) | 0.68 |
| **IPC 3: Explained results, medications** | -0.11 | 0.41 | -0.46 | (-0.70, -0.11) | 0.009 | 0.19 | (-0.23, 0.54) | 0.37 |
| **IPC 4: Patient-centered decision making^a^** | -0.06 | 0.68 | -0.24 | (-0.55, 0.13) | 0.20 | 0.13 | (-0.29, 0.50) | 0.56 |
| **HbA1c** | 0.14 | 0.29 | 0.25 | (-0.12, 0.56) | 0.18 | -0.02 | (-0.41, 0.38) | 0.92 |
| **Medication Adherence** | 0.04 | 0.75 | 0.20 | (-0.18, 0.53) | 0.30 | -0.17 | (-0.53, 0.25) | 0.43 |
|  | **Provider Self-Efficacy** | | | | | | | |
| **IPC 1: Hurried communication** | -0.22 | 0.11 | -0.43 | (-0.68, -0.07) | 0.018 | 0.02 | (-0.38, 0.41) | 0.93 |
| **IPC 2: Elicited concerns, responded** | 0.25 | 0.07 | 0.44 | (0.09, 0.69) | 0.013 | 0.14 | (-0.27, 0.51) | 0.50 |
| **IPC 3: Explained results, medications** | 0.28 | 0.038 | 0.46 | (0.11, 0.70) | 0.011 | 0.13 | (-0.28, 0.50) | 0.55 |
| **IPC 4: Patient-centered decision making^a^** | 0.23 | 0.09 | 0.31 | (-0.06, 0.60) | 0.09 | 0.15 | (-0.27, 0.52) | 0.48 |
| **HbA1c** | 0.02 | 0.87 | -0.16 | (-0.49, 0.21) | 0.39 | 0.32 | (-0.09, 0.63) | 0.12 |
| **Medication Adherence** | -0.10 | 0.49 | -0.13 | (-0.47, 0.25) | 0.50 | 0.04 | (-0.36, 0.43) | 0.86 |
| 1. See “Table 4: Overall Spearman Correlation Coefficients between PPRR and. Outcomes” for abbreviated results 2. N=54 due to nonresponse | | | | | | | | |

| **Supplemental Table 5: Linear Regression Analysis of PPRR vs. Outcomes** | | | | | | |
| --- | --- | --- | --- | --- | --- | --- |
|  | **Provider Belief** | | | | | |
|  | **Overall** | | | **Race Interaction** | | |
|  | **Beta** | **95% CI** | **p-value** | **Beta** | **95% CI** | **p-value** |
| **IPC 1: Hurried communication** | 0.13 | (-0.07, 0.33) | 0.19 | 0.33 | (-0.10, 0.75) | 0.13 |
| **IPC 2: Elicited concerns, responded** | -0.05 | (-0.23, 0.13) | 0.59 | -0.18 | (-0.58, 0.21) | 0.35 |
| **IPC 3: Explained results, medications** | -0.23 | (-0.54, 0.08) | 0.15 | -0.49 | (-1.16, 0.18) | 0.15 |
| **IPC 4: Patient-centered decision making** | -0.28 | (-0.68, 0.12) | 0.16 | -0.79 | (-1.64, 0.07) | 0.07 |
| **HbA1c** | 0.61 | (-0.15, 1.37) | 0.11 | 0.67 | (-0.99, 2.34) | 0.42 |
| **Medication Adherence** | 0.15 | (-0.31, 0.62) | 0.51 | 0.54 | (-0.47, 1.55) | 0.29 |
|  | **Provider Awareness** | | | | | |
| **IPC 1: Hurried communication** | 0.06 | (-0.10, 0.23) | 0.45 | 0.11 | (-0.23, 0.44) | 0.53 |
| **IPC 2: Elicited concerns, responded** | -0.04 | (-0.19, 0.11) | 0.58 | -0.05 | (-0.36, 0.25) | 0.74 |
| **IPC 3: Explained results, medications** | -0.07 | (-0.34, 0.19) | 0.58 | -0.45 | (-0.97, 0.07) | 0.09 |
| **IPC 4: Patient-centered decision making** | -0.09 | (-0.43, 0.26) | 0.61 | -0.18 | (-0.87, 0.51) | 0.60 |
| **HbA1c** | 0.55 | (-0.08, 1.19) | 0.09 | 0.29 | (-0.99, 1.57) | 0.66 |
| **Medication Adherence** | 0.11 | (-0.28, 0.50) | 0.59 | 0.58 | (-0.19, 1.35) | 0.14 |
|  | **Provider Self-Efficacy** | | | | | |
| **IPC 1: Hurried communication** | -0.22 | (-0.45, 0.01) | 0.07 | -0.36 | (-0.83, 0.11) | 0.13 |
| **IPC 2: Elicited concerns, responded** | 0.16 | (-0.05, 0.38) | 0.12 | 0.08 | (-0.37, 0.52) | 0.73 |
| **IPC 3: Explained results, medications** | 0.37 | (0.01, 0.74) | 0.045 | 0.04 | (-0.73, 0.80) | 0.93 |
| **IPC 4: Patient-centered decision making** | 0.43 | (-0.05, 0.90) | 0.08 | 0.19 | (-0.81, 1.18) | 0.71 |
| **HbA1c** | -0.15 | (-1.08, 0.78) | 0.75 | -1.37 | (-3.28, 0.54) | 0.16 |
| **Medication Adherence** | -0.09 | (-0.65, 0.46) | 0.74 | -0.27 | (-1.44, 0.89) | 0.64 |
