# Supplementary material for: Association of Provider Perspectives on Race and Racial Health Care Disparities with Patient Perceptions of Care and Health Outcomes
Source: Health Equity. 2021 Jul 5;5(1):466–75. doi: 10.1089/heq.2021.0018 (PMC8309434; doi:10.1089/heq.2021.0018)
Supplement: Supplemental data [file Supp_Table5.docx]

| **Supplemental Table 5: Linear Regression Analysis of PPRR vs. Outcomes** | | | | | | |
| --- | --- | --- | --- | --- | --- | --- |
|  | **Provider Belief** | | | | | |
|  | **Overall** | | | **Race Interaction** | | |
|  | **Beta** | **95% CI** | **p-value** | **Beta** | **95% CI** | **p-value** |
| **IPC 1: Hurried communication** | 0.13 | (-0.07, 0.33) | 0.19 | 0.33 | (-0.10, 0.75) | 0.13 |
| **IPC 2: Elicited concerns, responded** | -0.05 | (-0.23, 0.13) | 0.59 | -0.18 | (-0.58, 0.21) | 0.35 |
| **IPC 3: Explained results, medications** | -0.23 | (-0.54, 0.08) | 0.15 | -0.49 | (-1.16, 0.18) | 0.15 |
| **IPC 4: Patient-centered decision making** | -0.28 | (-0.68, 0.12) | 0.16 | -0.79 | (-1.64, 0.07) | 0.07 |
| **HbA1c** | 0.61 | (-0.15, 1.37) | 0.11 | 0.67 | (-0.99, 2.34) | 0.42 |
| **Medication Adherence** | 0.15 | (-0.31, 0.62) | 0.51 | 0.54 | (-0.47, 1.55) | 0.29 |
|  | **Provider Awareness** | | | | | |
| **IPC 1: Hurried communication** | 0.06 | (-0.10, 0.23) | 0.45 | 0.11 | (-0.23, 0.44) | 0.53 |
| **IPC 2: Elicited concerns, responded** | -0.04 | (-0.19, 0.11) | 0.58 | -0.05 | (-0.36, 0.25) | 0.74 |
| **IPC 3: Explained results, medications** | -0.07 | (-0.34, 0.19) | 0.58 | -0.45 | (-0.97, 0.07) | 0.09 |
| **IPC 4: Patient-centered decision making** | -0.09 | (-0.43, 0.26) | 0.61 | -0.18 | (-0.87, 0.51) | 0.60 |
| **HbA1c** | 0.55 | (-0.08, 1.19) | 0.09 | 0.29 | (-0.99, 1.57) | 0.66 |
| **Medication Adherence** | 0.11 | (-0.28, 0.50) | 0.59 | 0.58 | (-0.19, 1.35) | 0.14 |
|  | **Provider Self-Efficacy** | | | | | |
| **IPC 1: Hurried communication** | -0.22 | (-0.45, 0.01) | 0.07 | -0.36 | (-0.83, 0.11) | 0.13 |
| **IPC 2: Elicited concerns, responded** | 0.16 | (-0.05, 0.38) | 0.12 | 0.08 | (-0.37, 0.52) | 0.73 |
| **IPC 3: Explained results, medications** | 0.37 | (0.01, 0.74) | 0.045 | 0.04 | (-0.73, 0.80) | 0.93 |
| **IPC 4: Patient-centered decision making** | 0.43 | (-0.05, 0.90) | 0.08 | 0.19 | (-0.81, 1.18) | 0.71 |
| **HbA1c** | -0.15 | (-1.08, 0.78) | 0.75 | -1.37 | (-3.28, 0.54) | 0.16 |
| **Medication Adherence** | -0.09 | (-0.65, 0.46) | 0.74 | -0.27 | (-1.44, 0.89) | 0.64 |
